# Who uses emergency departments inappropriately and when - a national cross-sectional study using a monitoring data system

**DOI:** 10.1186/1741-7015-11-258

**Published:** 2013-12-13

**Authors:** Philip McHale, Sara Wood, Karen Hughes, Mark A Bellis, Ulf Demnitz, Sacha Wyke

**Affiliations:** 1 Centre for Public Health, Liverpool John Moores University, 15-21 Webster Street, Liverpool L3 2ET, UK; 2 Public Health Wales, Cardiff University, Hadyn Ellis Building, Maindy Road, Cardiff CF24 4HQ, UK; 3 Accident and Emergency, Royal Liverpool University Hospital, Prescot Street, Liverpool L7 8XP, UK; 4 Knowledge and Intelligence Team (North West), Public Health England, 15-21 Webster Street, Liverpool L3 2ET, UK

**Keywords:** Emergency department, Inappropriate attendance, Health service use

## Abstract

**Background:**

Increasing pressures on emergency departments (ED) are straining services and creating inefficiencies in service delivery worldwide. A potentially avoidable pressure is inappropriate attendances (IA); typically low urgency, self-referred patients better managed by other services. This study examines demographics and temporal trends associated with IA to help inform measures to address them.

**Methods:**

Using a national ED dataset, a cross-sectional examination of ED attendances in England from April 2011 to March 2012 (n = 15,056,095) was conducted. IA were defined as patients who were self-referred; were not attending a follow-up; received no investigation and either no treatment or ‘guidance/advice only’; and were discharged with either no follow-up or follow-up with primary care. Small, nationally representative areas were used to assign each attendance to a residential measure of deprivation. Multivariate analysis was used to predict relationships between IA, demographics (age, gender, deprivation) and temporal factors (day, month, hour, bank holiday, Christmas period).

**Results:**

Overall, 11.7% of attendances were categorized as inappropriate. IA peaked in early childhood (adjusted odds ratio (AOR) = 1.53 for both one and two year olds), and was elevated throughout late-teens and young adulthood, with odds reducing steadily from age 27 (reference category, age 40). Both IA and appropriate attendances (AA) were most frequent in the most deprived populations. However, relative to AA, those living in the least deprived areas had the highest odds of IA (AOR = 0.89 in most deprived quintile). Odds of IA were also higher for males (AOR = 0.95 in females). Both AA and IA were highest on Mondays, whilst weekends, bank holidays and the period between 8 am and 4 pm saw more IA relative to AA.

**Conclusions:**

Prevention of IA would be best targeted at parents of young children and at older youths/young adults, and during weekends and bank holidays. Service provision focusing on access to primary care and EDs serving the most deprived communities would have the most benefit. Improvements in coverage and data quality of the national ED dataset, and the addition of an appropriateness field, would make this dataset an effective monitoring tool to evaluate interventions addressing this issue.

## Background

Emergency departments (EDs) are an integral service for healthcare systems worldwide, providing immediate point-of-access care for urgent medical conditions and injuries. Despite this, across the globe, pressures and crowding are resulting in increasingly strained and inefficient ED services, leading to increased waiting times and treatment delays, impaired access, financial losses for providers, and ethical consequences [1,2]. A potentially avoidable part of increased pressures on ED services is ‘inappropriate’ attendances (IA); patients who self-refer with low urgency problems that are unlikely to require admission and are more suitable for other services, such as primary care, telephone advice helplines or pharmacy [3]. Wide variability exists when estimating the prevalence of these attendances, due in part to varying definitions and the subjective nature of measuring inappropriateness [4]. However, internationally, between 24% and 40% of all ED attendances are thought to be inappropriate [4]. Such IA can hinder the ability of EDs to treat attendees in a timely and consequently safe manner. Whilst low complexity patients may have a minimal effect on waiting times for more urgent attenders [5], non-urgent cases may equally prohibit access for real emergency cases [6] and have a negative impact on staff attitudes [7].

In England, IA to EDs are a long-standing problem [8]. Despite previous attempts to reduce their occurrence (for example, through advising people not to use EDs for non-urgent conditions and by providing a primary care service in EDs [9-11]), IA are thought to remain a burden on ED services. One local study of patients from two health centers attending a single ED found that 16.8% of attendances were inappropriate [12], while a broader study of ED use in one London borough reported that 78% of all attendances were potentially avoidable [13]. Across England, increasing attendance figures and insufficient staffing levels are placing the ED system at crisis point, with hospital trusts increasingly failing to meet four-hour waiting time targets [14-16]. With an urgent need to reduce current burdens on ED services, developing and targeting interventions to reduce or manage levels of IA should be a priority. As a first step to achieving this, it is necessary to gain a good understanding of the burden IA place on EDs, the types of people most likely to present inappropriately, and when such attendances are most likely to occur.

When previous studies of inappropriate or avoidable ED attendances in England have been conducted [12,13], they have focused on single or multiple hospitals in local areas. This study explores IA across England as a whole. Since 2007, records of attendance to National Health Service EDs in England have been recorded into an experimental dataset using the Hospital Episode Statistics (HES) system. This study utilizes these data to determine the current prevalence of IA in England and details the demographic and temporal profiles of these attendances.

## Methods

We extracted data from the HES A&E system, for all attendances to ED between 1 April 2011 to 31 March 2012 (n = 17,470,479). The HES A&E system includes major EDs (that is, consultant led, permanently open and with full resuscitation facilities), specialty EDs, walk-in centers and minor injury units. The 2011 to 2012 dataset is estimated to include 80.5% of all English ED attendances [17]. Extracted variables included: attendee age, gender and area of residence; date and time of arrival; attendance disposal; attendance category; department type; primary investigation; and primary treatment. We excluded attendances to National Health Service walk-in center departments (n = 912,167); those with missing or unknown gender (n = 191,262); those with unknown age, missing age or an age likely to be unreliable through extreme old age (≥110 years; n = 9,901); those with missing area of residence (n = 171,427); those with attendance category not known (n = 2,727); those with patient group brought in dead (n = 1,969); and those with attendance disposal category ‘left before being treated’, ‘left having refused treatment’, or missing (n = 650,848). The remaining sample was 15,530,178.

Attendances were mapped to area of residence by lower super output area in the HES data. Lower super output areas are a standard geography of mean population 1,500, and each is assigned a ranking of deprivation by the Index of Multiple Deprivation, a collection of indicators across seven domains of deprivation. Thus, each attendance was assigned to a national ecological measure of deprivation based on their lower super output area of residence [18].

Attendances were assigned to two groups of appropriateness: appropriate attendances (AA) and IA. Attendances were assigned to AA if the source of referral was any other than self-referred; attendance category was planned follow-up; the attendance had a valid investigation code other than ‘none’, or a valid treatment code other than ‘none’ or ‘guidance/advice only’; and disposal method was either admission, referral to clinic, transfer to other healthcare provider, referral to other healthcare professional or other. Attendances were assigned to IA if the source of referral was self-referred; attendance category was the initial ED attendance or unplanned follow-up; investigation code was ‘none’ and treatment code was either ‘none’ or ‘guidance/advice only’; and disposal method was discharge with no follow-up or discharge with follow-up from general practitioner. Attendances that did not match these criteria were excluded (3.1% of remaining sample). The final sample size was 15,056,095.

Data were analyzed in Predictive Analytics Software (PASW®) v19 (International Business Machines Corporation, Statistical Package for the Social Sciences, Armonk, New York, USA). Initial analysis of demography and time variables was performed using chi-squared on the two groups of appropriateness. Generalized linear modeling was used to calculate estimated marginal means for weekday and month. Estimated marginal means are used when average values require correcting for the impact of other confounding variables. Backward conditional binary logistic regression was used to calculate adjusted odds for IA compared with AA by demographic variables and time variables separately.

Ethical approval for this study was obtained from Liverpool John Moores University Research Ethics Committee.

## Results

Of the 15,056,095 attendances included in the analysis (86.2% of all recorded attendances), 88.3% (13,294,819) were categorized as AA and 11.7% (1,761,276) as IA. Rates of IA per 100 attendances were highest in those under 16 years of age and lowest in those over 85 (15.04 and 2.43 per 100 attendances respectively; *P*<0.001; Table 1), and were higher in males than females (12.26 and 11.12 per 100 attendances respectively; *P*<0.001; Table 1). As deprivation increased, the total number of both AA and IA also increased. For IA, this increase was from 256,624 in the least deprived quintile to 474,652 in the most deprived quintile. However, no distinct relationship existed when considering IA rates per 100 attendances, with the second most deprived quintile having the highest rate and the fourth most deprived the lowest (12.03 and 11.50 respectively; *P*<0.001; Table 1). The highest proportion of attendances in both IA and AA groups was made up of individuals who only attended the ED once in the time period (46.6% in AA; 53.9% in IA). The rate of IA was highest in single attendances and lowest in individuals who attended 11 to 20 times (13.28 and 7.41 respectively; *P*<0.001; Table 1).

**Table 1:** Number, distribution (%) and rate of appropriate and inappropriate emergency department attendances by demographics and repeat attendances

	**Appropriate**		**Inappropriate**		**Rate of inappropriate attendances per 100 attendances**	***P***
	**n = 13,294,819**		**n = 1,761,276**			
	**n**	**%**	**n**	**%**		
**Age group (years)**						
0 to 15	2,770,259	20.84	490,360	27.84	15.04	
16 to 24	1,823,858	13.72	293,820	16.68	13.87	
25 to 39	2,504,987	18.84	399,913	22.71	13.77	
40 to 54	2,165,611	16.29	301,399	17.11	12.22	
55 to 64	1,127,313	8.48	123,346	7.00	9.86	
65 to 84	2,145,985	16.14	133,584	7.58	5.86	
85 plus	756,806	5.69	18,854	1.07	2.43	<0.001
**Gender**						
Male	6,714,111	50.50	938,131	53.26	12.26	
Female	6,580,708	49.50	823,145	46.74	11.12	<0.001
**IMD quintile**						
Least deprived	1,955,886	14.71	256,624	14.57	11.60	
Fourth most deprived	2,199,109	16.54	285,731	16.22	11.50	
Third most deprived	2,539,095	19.10	335,480	19.05	11.67	
Second most deprived	2,989,953	22.49	408,789	23.21	12.03	
Most deprived	3,610,776	27.16	474,652	26.95	11.62	<0.001
**Repeat attendances**^**a**^						
1	6,195,506	46.60	948,869	53.87	13.28	
2	3,267,400	24.58	422,056	23.96	11.44	
3	1,589,839	11.96	175,976	9.99	9.97	
4	824,571	6.20	86,701	4.92	9.51	
5	450,448	3.39	42,387	2.41	8.60	
6 to 10	674,692	5.07	59,888	3.40	8.15	
11 to 20	193,523	1.46	15,484	0.88	7.41	
21 to 50	77,540	0.58	7,224	0.41	8.52	
51 or more	21,300	0.16	2,691	0.15	11.22	<0.001

^**a**^Repeat attendances are individuals who attend the emergency department numerous times, not including planned follow-up attendances. IMD, Index of Multiple Deprivation.

**Figure 1:**
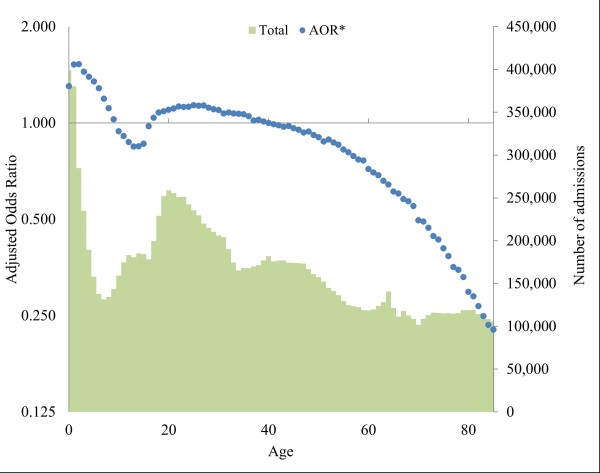
**Adjusted odds ratios (reference age 40) and total attendances by year of age.** Ages above 85 not included due to small totals. Confidence intervals were too small to be displayed. Logistic regression model controlled for age, gender and deprivation. Primary Y axis is a logarithmic scale (base 2). *Adjusted odds ratio.

In multivariate analysis, relationships between IA and both age and gender remained similar, whereas the relationship with deprivation reversed compared with total attendances. Females were less likely to attend inappropriately than males (adjusted odds ratio (AOR) = 0.95; *P*<0.001), whilst odds of IA decreased with increasing deprivation, with residents from the most deprived quintile having the lowest odds of IA (AOR = 0.89; *P*<0.001; Table 2). Age showed a strong relationship with IA (Figure 1). Adjusted odds peaked at ages one and two years (both AOR = 1.53; *P*<0.001), then fell through to age 13 (AOR = 0.84; *P*<0.001), before rising again to age 25 (AOR = 1.14; *P*<0.001). After this age, there was a steady fall as age increased.

**Table 2:** Adjusted odds ratios for inappropriate attendances

		**Adjusted odds ratio**	**95% confidence interval**		***P***
			**Lower**	**Upper**	
**Logistic regression A**^**a**^					
**Gender**	Male (Ref)	1.00	-	-	
	Female	0.95	0.95	0.95	<0.001
**IMD quintile**	Least deprived (Ref)	1.00	-	-	
	Fourth most deprived	0.99	0.98	0.99	<0.001
	Third most deprived	0.98	0.97	0.98	<0.001
	Second most deprived	0.96	0.96	0.97	<0.001
	Most deprived	0.89	0.89	0.90	<0.001
**Logistic regression B**^**b**^					
**Month**	June (Ref)	1.00	-	-	
	July	1.04	1.04	1.05	<0.001
	August	1.04	1.03	1.05	<0.001
	September	1.01	1.00	1.01	NS
	October	0.99	0.98	0.99	<0.01
	November	0.98	0.97	0.98	<0.001
	December	0.97	0.96	0.98	<0.001
	January	0.97	0.96	0.98	<0.001
	February	0.98	0.97	0.99	<0.001
	March	0.99	0.99	1.00	NS
	April	0.99	0.98	1.00	<0.05
	May	0.97	0.96	0.97	<0.001
**Weekday**	Wednesday (Ref)	1.00	-	-	
	Thursday	0.99	0.99	1.00	<0.001
	Friday	0.97	0.96	0.98	<0.001
	Saturday	1.10	1.09	1.11	<0.001
	Sunday	1.09	1.09	1.10	<0.001
	Monday	1.02	1.01	1.03	<0.001
	Tuesday	1.00	0.99	1.01	NS
**Special Periods**	Bank Holiday	1.13	1.12	1.14	<0.001
	Christmas Period	0.97	0.94	0.99	<0.01

**Figure 2:**
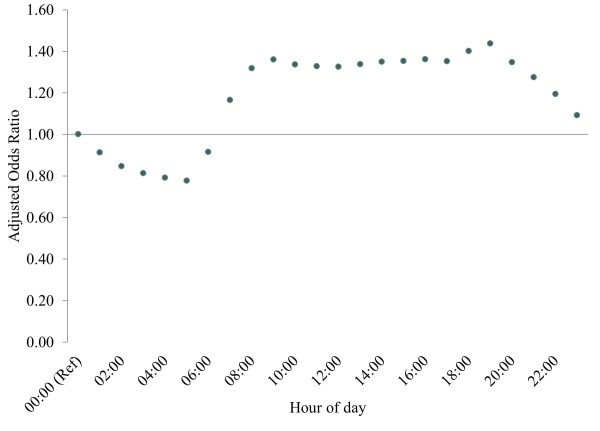
**Adjusted odds ratios for hour of arrival (reference hour is midnight).** Confidence intervals were too small to be displayed. Logistic regression model controlled for hour of arrival, month, weekday, bank holiday and Christmas period.

Two separate logistic regression models were used. ^a^Variables controlled for in regression A were gender, deprivation and age (adjusted odds ratio for year of age is included in Figure 1). ^b^Variables controlled for in regression B were month, weekday, hour^†^, bank holidays and Christmas period (AOR for hour is included in Figure 2). IMD, Index of Multiple Deprivation.

Table 3 shows the estimated marginal means of daily admissions by weekday and month. Estimated marginal means of daily attendances were highest for both AA and IA in March (39,033 and 5,166 respectively) and lowest in August and December for AA (34,255 and 34,996 respectively) and in January and December for IA (4,564 and 4,502 respectively). Rate of IA was highest in August and lowest in December (12.20 and 11.40 respectively; *P*<0.001). Monday had the highest estimated marginal means of daily attendance for both AA and IA (40,290 and 5,408 respectively) while Friday had the lowest for IA (4,465) and Saturday the lowest for AA (34,475). Rates of IA were highest on Saturday and lowest on Friday (12.31 and 11.17 respectively; *P*<0.001). Both AA and IA were lowest between midnight and 8 am, whilst they were highest between 8 am and 4 pm (Table 4). Rates of IA were highest between 8 am and 4 pm, and lowest between midnight and 8 am (12.09 and 8.71 respectively; *P*<0.001).

**Table 3:** Estimated marginal means for daily attendances by month and weekday

		**Appropriate**		**Inappropriate**		**Rate of inappropriate attendances per 100 attendances**
		**n = 13,294,819**		**n = 1,761,276**		
		**Mean**	**EMM**	**Mean**	**EMM**	
**Month**	January	35,359	35,238	4,587	4,564	11.47
	February	36,928	36,945	4,813	4,819	11.54
	March	38,927	39,033	5,148	5,166	11.69
	April	36,343	36,433	4,852	4,863	11.78
	May	36,527	36,406	4,750	4,727	11.49
	June	35,797	35,832	4,748	4,761	11.73
	July	36,191	36,297	5,033	5,038	12.19
	August	34,350	34,225	4,768	4,756	12.20
	September	36,746	36,793	4,910	4,929	11.81
	October	37,351	37,303	4,923	4,898	11.61
	November	36,529	36,532	4,731	4,738	11.48
	December	34,891	34,996	4,484	4,502	11.40
	*P*	<0.001	<0.001	<0.001	<0.001	<0.001
**Weekday**	Sunday	35,728	35,730	4,976	4,977	12.23
	Monday	40,248	40,290	5,402	5,408	11.83
	Tuesday	36,687	36,743	4,764	4,773	11.50
	Wednesday	35,820	35,855	4,642	4,645	11.47
	Thursday	35,771	35,746	4,588	4,586	11.37
	Friday	35,548	35,513	4,473	4,465	11.17
	Saturday	34,519	34,475	4,846	4,839	12.31
	*P*	<0.001	<0.001	<0.001	<0.001	<0.001

EMM, estimated marginal mean.

**Table 4:** Number of inappropriate and appropriate attendances, and rate of inappropriate attendance by eight-hour time period

	**Appropriate**	**Inappropriate**	**Rate of inappropriate attendance per 100 attendances**	***P***
	**n = 13,294,819**	**n = 1,761,276**		
00:00 to 07:59	1,513,580	144,416	8.71	
08:00 to 15:59	6,655,458	915,453	12.09	
16:00 to 23:59	5,125,781	701,407	12.04	<0.001

After controlling for various time periods of attendance (hour, month, weekday, bank (public) holiday and Christmas period; Table 2), IA had the highest AOR during the months of July and August (both AOR = 1.04; *P*<0.001), on Saturdays (AOR = 1.10; *P*<0.001), and on bank holidays (AOR = 1.13; *P*<0.001). By contrast, odds of IA were lowest in the months of May and December (both AOR = 0.97; *P*<0.001), on Fridays (AOR = 0.97; *P*<0.001), and during the Christmas period (AOR = 0.97; *P*<0.001) (Table 3). IA were lowest between midnight and 6 am (lowest 5 am, AOR = 0.78; *P*<0.001) with attendances increasing after this point to plateau between 9 am and 7 pm (highest 7 pm, AOR = 1.44; *P* <0.001), then fall again to midnight (Figure 2).

## Discussion

IA are a long-standing concern for EDs and still provide an excess burden on services [13]. This ecological study provides insight into the demographical representation and temporal factors associated with IA to ED. The large sample has allowed for a level of analysis that has not been previously performed. Almost 12% of the attendances in our study were deemed inappropriate, a value substantially lower than others reported internationally [4]. This marked difference is likely due to the definition of IA used. Our retrospective definition includes only attendances that were self-referred, received no investigation and either advice or no treatment, and were discharged without any follow-up or to primary care. These are easily measurable criteria likely to provide high specificity, but by using a generic definition of IA, it is unavoidable that certain individual attendances can be misallocated. For example, attendees who receive simple medication (for example, over-the-counter analgesia) that could readily be provided by other services (like pharmacy) will be grouped as appropriate, whereas attendees with certain psychiatric complaints may require emergency assessment and no other investigation or intervention, and thus be deemed inappropriate.

Reducing IA to EDs could have a significant effect on the quality and continuity of care provided to patients, and also on the overall financial cost of this service. Using our findings, IA resulted in an estimated cost of nearly £100 million between April 2011 and March 2012, assuming an ED attendance with no investigation or significant treatment cost £54 [19]. It should be noted, however, that many costs of EDs are relatively fixed (such as staffing and opening of a department), and further research would be required to examine whether reductions in IA would result in cost saving or increased efficiency and utility of existing resources.

We found age to have a strong relationship with IA. Odds of IA were highest in the very young (peak attendances were in one and two year olds), and elevated between mid-teens and mid-twenties, followed by a steady fall as age increased thereafter. The inverse relationship between IA and age found in our study has also been identified elsewhere (for example, USA, Canada and Brazil) [20-22]. Thus, our findings suggest that interventions to prevent IA should be targeted towards early childhood and young adults in their late teens to late twenties. For young children, the decision to attend ED lies with parents and guardians, and this is likely reflective of the pressures of parenthood and a belief that the ED is the most appropriate place to receive care [23,24]. This could be offset through targeted education to parents about the appropriate use of ED services, or by providing details of other local health services capable of providing prompt medical advice when to access to primary care and out-of-hours services available. This could be delivered routinely via health professionals who have a high degree of contact with new parents (for example, through home visits and routine health checks), such as health visitors, community midwives and nursery nurses.

The second peak in odds of IA seen from late teens to late twenties could reflect a time when young people are leaving home for the first time (such as to attend university), and may indicate a poor understanding regarding appropriate use of ED services, a lack of knowledge of other health services available, and poor access to primary care (for example, still registered with childhood general practice). Targeted education for school leavers and university students regarding appropriate use of ED, alternative health services available in the local area and the importance of primary care and registration with a local general practitioner could prove useful in reducing IA in this group.

Research in other countries has found that females are more likely to attend ED inappropriately than males (for example, Brazil, Turkey and USA [22,25,26]). Conversely, we found that males were slightly more likely to attend inappropriately than females, although absolute differences were small. This may reflect a difference in the definition of IA or differences in the structure and use of health services between countries. Whilst deprivation has been linked to IA in other studies, the direction of association has been mixed and may depend to some extent on the marker of deprivation used (for example, education, income, social class or postcode) [13,25,27]. Using a measure of residential deprivation, we found that the most deprived population accounted for the highest numbers of both AA and IA. This likely represents the poorer health and greater injury risk experienced in deprived communities. However, after controlling for age and gender, those from the least deprived quintile had the greatest odds of IA relative to AA. Several mechanisms might explain this finding, including greater access to ED among more affluent individuals (such as through increased availability of transport) [28], and smaller family size possibly permitting greater focus on individual children and increased concern over non-urgent conditions [23,29]. There is a need for greater clarification around this relationship to help understand why certain social groups may be more likely to attend inappropriately than others. Although significant, the degree of difference between deprivation quintiles is only small and with IA occurring most often in the most deprived communities, measures to manage service pressures by providing additional services and addressing IA would be of greatest benefit in deprived areas.

Both AA and IA were seen to occur most regularly on Mondays, during March and between 8 am and 4 pm. When controlling for temporal effects, relative to AA odds of IA were significantly higher on weekend days, bank holidays and between the hours of 8 am and midnight. These findings can inform both the management of ED services and prevention of IA; service provision may be best targeted on Mondays and between the hours of 8 am and 4 pm, whilst measures to raise awareness may be most effective if targeted at weekends and Bank Holidays. The increase during weekends and bank holidays likely represents a lack of access to primary care during these times, and a reluctance to take time off work during the week to access these services. Although significant, the variation seen in IA by month was smaller than that seen when comparing weekdays to weekends or bank holidays, suggesting that month is unlikely to be a major factor informing service management or prevention measures regarding IA.

Internationally, numerous methods have been used to prevent inappropriate use of EDs. These include diverting calls from emergency services, ambulance non conveyance, attempts to triage out IA and general education [30]. These interventions have experienced variable success and have raised questions over patient safety [30]. Patient safety is paramount and any potential negative effects of intervention (for example, a delay in attendance to an ED for an urgent health problem) must be carefully considered before implementation. A further method of addressing IA, trialed in England, is the provision of primary care physicians either alongside emergency physicians in the ED itself, or attached to the department in a general practice surgery [11]. This is intended to provide alternative options for what is deemed an IA at ED, with research suggesting it is a safe and cost-effective intervention [31,32], and one supported by the College of Emergency Medicine [33]. It has been suggested that primary care services are currently insufficient to manage the demand for health treatment and require modification to reduce the burden on ED [16].

The major strength of our study is its scope; the HES A&E dataset has provided access to a much larger sample of IA than previously studied and one that represents the majority of ED attendances in England over a one-year period. Also, the use of the Index of Multiple Deprivation has allowed for a more comprehensive review of deprivation comparative to previous literature. However, a number of limitations do exist. Firstly, alongside potential misallocation of attendances to either the IA or AA groups, only using attendances that were self-referred will have missed any inappropriate cases referred from primary care, telephone triage services or the ambulance service, while the exclusion of cases who left the ED before being treated or having refused treatment may have further missed IA. We were also unable to account for variation in staff practices regarding investigation and treatment. Despite this, our definition should act as a suitable proxy for IA, and results will remain relevant when considering prevention or management. Another limitation is the lack of additional data which is inevitable when using datasets such as the HES. Information on access to primary care services, reasons for choosing ED as point of care, general impressions of different services and patients’ own view of attendance appropriateness will be important in determining potential predictors of IA. Additionally, the incompleteness of the dataset is an important limitation. Although small relative to our sample size, over 470,000 attendances were removed because they could not be assigned to an appropriateness category. In addition, only 62.6% of attendances had a valid diagnosis code [17], preventing analysis of these data, which would have provided information that could further inform prevention.

This study is the first to explore IA across England as a whole using the HES dataset for ED attendances. Whilst this dataset is currently experimental, coverage across England continues to improve each year, with 80.5% of all ED attendances in England included in data for 2011 to 2012 and over 90% of cases having valid data on investigation, treatments and disposal [17]. With an urgent need to reduce the burden on ED across England, this dataset, and the methods detailed in our study, could readily form the basis of a monitoring system, allowing for timely evaluation of interventions and services implemented to alleviate the ED burden of IA and increase the quality of the service. To strengthen the dataset, an appropriateness field could be added, which could alleviate concerns about sensitivity. To do this, a clear definition of IA would be required, based on objective criteria rather than subjective evaluation. Such a definition of IA must be highly robust and exclude any attendance with a risk of serious sequelae resulting from non-use of ED. A national policy for clinical ED staff to determine appropriateness via these criteria would allow for effective inclusion of IA into the HES A&E dataset.

## Conclusions

Our study adds important evidence to the field of ED attendance research. The clear relationship between IA and age indicates that prevention would be best targeted at parents of young children (age under 10 years) and at young adults (aged 20 to 29). Increased odds of attendance on weekends and bank holidays, and in younger age groups, suggests that reduced access to primary care is an important factor in IA. The methods used here could form the basis of a monitoring system to evaluate the effectiveness of any interventions implemented to reduce IA. These results will be useful in creating policies to either reduce or manage the current burden of IA on emergency services.

## Abbreviations

A&E: Accident and emergency; AA: Appropriate attendances; AOR: Adjusted odds ratio; ED: Emergency department; HES: Hospital Episode Statistics; IA: Inappropriate attendances.

## Competing interests

The authors declare that they have no competing interests.

## Authors’ contributions

MAB and PM designed the study. PM analyzed the data and drafted the manuscript. SW co-drafted the manuscript and contributed to data analysis. MAB and KH contributed to data analysis and manuscript editing. UD contributed to manuscript editing. SWy extracted the data. All authors read and approved the final manuscript.

## Pre-publication history

The pre-publication history for this paper can be accessed here:

http://www.biomedcentral.com/1741-7015/11/258/prepub
